# A recombinant rabies virus chimera expressing the DC-targeting molecular MAB2560 shows enhanced vaccine immunogenicity through activation of dendritic cells

**DOI:** 10.1371/journal.pntd.0011254

**Published:** 2023-04-24

**Authors:** Zhiyuan Gong, Pei Huang, Hongli Jin, Yujie Bai, Hailun Li, Meichen Qian, Jingxuan Sun, Cuicui Jiao, Mengyao Zhang, Yuanyuan Li, Haili Zhang, Hualei Wang

**Affiliations:** 1 Key Laboratory of Zoonosis Research, Ministry of Education, College of Veterinary Medicine, Jilin University, Changchun, China; 2 Changchun Sino Biotechnology Co., Ltd., Changchun, China; University of São Paulo, BRAZIL

## Abstract

**Background:**

Rabies, caused by the rabies virus (RABV), is an ancient and neglected zoonotic disease posing a large public health threat to humans and animals in developing countries. Immunization of animals with a rabies vaccine is the most effective way to control the epidemic and the occurrence of the disease in humans. Therefore, the development of cost-effective and efficient rabies vaccines is urgently needed. The activation of dendritic cells (DCs) is known to play an important role in improving the host immune response induced by rabies vaccines.

**Methodology/Principal findings:**

In this study, we constructed a recombinant virus, rCVS11-MAB2560, based on the reverse genetic system of the RABV CVS11 strain. The MAB2560 protein (a DC-targeting molecular) was chimeric expressed on the surface of the viral particles to help target and activate the DCs when this virus was used as inactivated vaccine. Our results demonstrated that inactivated rCVS11-MAB2560 was able to promote the recruitment and/or proliferation of DC cells, T cells and B cells in mice, and induce good immune memory after two immunizations. Moreover, the inactivated recombinant virus rCVS11-MAB2560 could produce higher levels of virus-neutralizing antibodies (VNAs) in both mice and dogs more quickly than rCVS11 post immunization.

**Conclusions/Significance:**

In summary, the recombinant virus rCVS11-MAB2560 chimeric-expressing the molecular adjuvant MAB2560 can stimulate high levels of humoral and cellular immune responses in vivo and can be used as an effective inactivated rabies vaccine candidate.

## Author summary

Due to the lack of effective treatments and drugs, rabies is still an important zoonotic disease, posing a great risk to animals and human public health in developing countries. Vaccines, especially inactivated vaccines, are currently the most effective way to prevent and control the rabies epidemic. Here, we constructed a recombinant virus rCVS11-MAB2560 chimeric-expressed MAB2560 protein (a DC-targeting molecular) on the surface of the RABV particles to promote its immunogenicity in vivo by activating the dendritic cells (DCs) and enhancing antigen uptake. Our results showed that inactivated rCVS11-MAB2560 was able to promote the recruitment and/or proliferation of DC cells, T cells and B cells in mice, and induce good immune memory after two immunizations. Moreover, compared with rCVS11, the inactivated recombinant virus rCVS11-MAB2560 could induce higher level of virus-neutralizing antibodies (VNAs) in mice and dogs more quickly post immunization. rCVS11-MAB2560 thus has potential to be used as a promising candidate for an effective inactivated rabies vaccine.

## Introduction

Rabies is an ancient zoonotic disease in humans and almost all other mammals, caused by a neuronophagic virus, the rabies virus (RABV). The disease has a fatality close to 100% once clinical symptoms have developed, and is thus regarded as a major threat to global public health [1]. Although the epidemic of rabies has been controlled in developed countries and regions [2,3], its prevalence is maintained at a high level in many developing countries, particularly in Africa and Asia [4–12]. In these areas, rabies dogs are the main source of infection of the disease in humans. The World Health Organization (WHO) estimates that rabies causes approximately 59 thousand human deaths worldwide every year [13]. Although the case fatality is high and effective drugs to treat rabies are lacking, the disease is still preventable in humans and animals through vaccination [14–16]. The first rabies vaccine was developed in 1885 by Pasteur and was used for the treatment of rabies. Subsequently, several other rabies vaccines were developed and deployed, including inactivated vaccines [17], live attenuated vaccines [18], adenovirus vector vaccines [19], and others. Inactivated vaccines are currently used worldwide as the most effective means to control rabies in humans. However, inactivated vaccines do have certain disadvantages. In addition to side effects caused by aluminum adjuvants, the cumbersome immunization procedure by which the inactivated vaccines are administered (3 times pre-exposure or 4 to 5 times post-exposure in humans) [20] poses a large economic burden on individuals and society, and sometimes leads to immunization failure due to incompletion of the immunization procedure. There is therefore an urgent need for cheap and highly effective vaccines that can induce antibodies quickly and efficiently, to help achieve the goal of elimination of dog-mediated human rabies deaths by 2030 [21].

Recently, it has been suggested that the activation of dendritic cells (DCs) and subsequent signaling pathways is critical for antigen uptake and for the vaccine-induced immune response against rabies [22,23]. Many studies have shown that overexpression of cytokines including murine granulocyte-macrophage colony-stimulating factor (GM-CSF) [24], IL-15 [25], CCL-3 (MIP-1α) [26], high mobility group box 1 (HMGB1) [27], or fms-like tyrosine kinase 3 ligand (Flt3L) [28] in recombinant RABV can enhance the humoral immune response by inducing the activation of DCs and promoting substantial VNA production. Certain other cytokines/chemokines, including CXCL-13 [29], interleukin-21 (IL-21) [30], and costimulatory factor OX40-ligand (OX40L) [31], are also able to contribute to the humoral immune response when overexpressed by recombinant RABV, by increasing the number of T follicular helper cells and germinal center B cells. However, in the studies mentioned above, overexpression of cytokines relied on the replication of the recombinant RABVs in host cells.

In this study, we employed a chimeric strategy to express a cytokine as a molecular adjuvant on the surface of viral particles, to promote the immunogenicity of an inactivated rabies vaccine in vivo. MAB2560, a noncytotoxic *Mycobacterium abscessu*-encoded nonstructural protein consisting of 201 amino acids, can activate DCs via the TLR4-mediated mitogen-activated protein kinase (MAPK) pathway, thus having potential as a Th1 polarizing molecular adjuvant [32]. Here, we constructed a recombinant RABV chimeric expressing the MAB2560 to enhance antigen uptake and promote the immune response, and we evaluated its immunogenicity in mice and dogs.

## Materials and methods

### Ethics statement

All of the mice and dogs used in this study were treated following the Chinese ethical guidelines for the welfare of laboratory animals (GB 14925–2010). The study was approved by the Animal Welfare and Ethics Committee of Jilin University (Laboratory Animal Care and Use Committee Authorization permit number SY202207010).

### Plasmids, cells, and antibodies

The plasmid pCDNA3.0-CVS11 containing the full-length infectious cDNA of CVS11 was constructed previously by our lab. This plasmid can enable foreign gene insertion between the *G* and *L* genes using the double restriction sites *BsiW* I and *Sac* II. The helper plasmids required for the rescue of recombinant viruses were kept in our lab, and included pD-N, pD-P, pD-L, and pD-G [33]. The plasmids pD-N, pD-P and pD-L were used to assemble the RNA polymerase of RABV and trigger the replication of the full-length infectious cDNA of RABV. The plasmid pD-G expressing RABV G protein was objected to help promoting the packing efficiency of progeny virions. Mouse neuroblastoma N2A (NA) cells, hamster kidney (BHK21) cells, and BSR cells (derived from BHK-21 cells expressing T7 RNA polymerase) were cultured in Dulbecco’s modified Eagle’s medium (DMEM, Gibco, USA) containing 10% fetal bovine serum (FBS, Gibco, USA). A fluorescein isothiocyanate (FITC)-conjugated monoclonal antibody (mAb) against the RABV N protein (800–092) was purchased from Fujirebio (Melvin, USA). The TRITC-conjugated goat anti-mouse IgG (T5393) was purchased from Sigma (St. Louis, USA). The mouse anti-RABV G mAb (MAB8727) and FITC-anti-rabbit antibody were purchased from Millipore (Billerica, USA). Flow cytometry antibodies, including PE-Cy7 labeled CD11c antibody and FITC labeled MHC-I (H-2KD) antibody, were purchased from the BD Pharmingen company. Rabbit anti-MAB2560 polyclonal antibody was prepared by our lab. Briefly, the recombinant MAB2560 proteins were expressed by prokaryotic system and purified by His-tag (Ni Sepharose 6 Fast Flow). The rabbits (12-weeks-old) were immunized twice with the purified MAB2560 proteins at 2-week intervals. The rabbit bloods were collected at 5 weeks after the first immunization to prepare polyclonal antibody against MAB2560.

### Construction of the recombinant virus rCVS11-MAB2560

The coding region of *MAB2560* gene (82–603 bp encoding 174 amino acid residues, excluded its signal peptide sequences) were amplified by PCR. The signal peptide and the TMCD domain of the G protein from the RABV CVS11 strain were ligated to the 3’ and 5’ ends of this product using PCR, respectively. The fragment was then inserted into the pCDNA3.0-CVS11 plasmid using the *BsiW* I and *Sac* II restriction sites, to construct the plasmid pCDNA3.0-CVS11-SP-MAB2560-TMCD. The recombinant pCDNA3.0-CVS11-SP-MAB2560-TMCD and pCDNA3.0-CVS11 plasmids were co-transfected into BSR cells with the helper plasmids to rescue the recombinant rCVS11-MAB2560 and rCVS11 viruses [33, 34]. Briefly, BSR cells were seeded into 6-well plate (3×10^5^/mL) and were transfected with the recombinant plasmids (2.5μg), the helper plasmids pD-N (0.625μg), pD-P (0.3125μg), pD-L (0.125μg), and pD-G (0.1875μg). After 72 h, the supernatants were collected for direct immunofluorescence assay (DFA).

### Indirect Immunofluorescence Assay (IFA)

NA cells were seeded into 96-well plates (2×10^5^/mL) and were infected with rCVS11-MAB2560 and rCVS11 at an MOI of 0.1. The cells were cultured in a 5% CO_2_ incubator at 37°C for 48 h. The cells were then fixed with 80% ice-cold acetone, and stained with rabbit anti-MAB2560 polyclonal antibody (1:200) at 37°C for 1 h. FITC-anti-rabbit antibody (1:300) was added and the samples were incubated at 37°C for 1 h. The fluorescent signals were then assessed using an inverted fluorescence microscope.

### Confocal microscopy

NA cells were seeded into a 24-well plate (2.5×10^4^/mL) and infected with either rCVS11-MAB2560 or rCVS11 at an MOI of 0.4. The cells were cultured in a 5% CO_2_ incubator at 37°C for 48 h. Samples were then fixed with 4% paraformaldehyde at 4°C, and blocked with 1% BSA at room temperature (RT) for 30 min. Rabbit anti-MAB2560 polyclonal antibody (1:200) and mouse anti-RABV G mAb (1:500) were used as primary antibodies. The samples were then incubated with FITC-labeled anti-rabbit secondary antibody (1:300) and TRITC-labeled anti-mouse secondary antibody (1:500) at RT for 1 h. The cells were then stained with DAPI anti-fluorescence quencher and assessed using laser confocal microscopy.

### Inactivation and purification of recombinant viruses

The viruses were mixed with β-propiolactone (1:2000) and inactivated at 4°C for 24 h. The inactivated viruses were centrifuged at 3000 rpm at 4°C for 30 min and the supernatants were then collected and mixed with 1M zinc acetate at 1/50 volume and kept at 4°C for 30 min. The precipitate was collected by centrifuging the samples at 12000 rpm for 30 min and was then resuspended in an appropriate amount of saturated EDTA. The mixture was purified using sucrose gradient centrifugation (20%, 30%, 40%, 55% sucrose). The samples retained in 30–40% sucrose were collected and resuspended in STE buffer (pH = 7.4, 0.15 M NaCl, 0.001 M EDTA and 0.01 M Tris). Quantification of purified viruses was determined using the BCA method.

### Western Blotting (WB)

The purified recombinant rCVS11-MAB2560 and rCVS11 viruses were quantified, and equal amounts (50μg) of each virus were subjected to SDS-PAGE electrophoresis. The gels were then transferred onto nitrocellulose (NC) membranes. After blocking with 5% milk in PBST (0.05% Tween20 in 1×PBS) at RT for 2h, the membranes were incubated with mouse anti-RABV G mAb (1:500) or rabbit anti-MAB2560 polyclonal antibody (1:200) overnight at 4°C. The membranes were then incubated with HRP-conjugated goat anti-mouse IgG antibody (1:10000) or HRP-conjugated goat anti-rabbit IgG antibody (1:20000) for 1h at RT. Electrochemiluminescence (ECL) Western Blotting Substrate (Pierce, USA) was then added, and the membranes were analyzed using a Tanon-5200 Chemiluminescent imaging system (Tanon, China).

### Growth curves and genetic stability

The recombinant rCVS11-MAB2560 and rCVS11 viruses were inoculated separately into NA cells or BSR cells at MOI = 1 or 0.1. The supernatants were collected every 24 h. Virus titer was determined using the Reed-Muench method as described previously [34]. The neural tropism of the recombinant viruses was calculated by comparing the titers of rCVS11-MAB2560 or rCVS11 in NA cells (a kind of neurogenic cells) to those of in BSR cells (non-neurogenic cells) and represented as the ratio index. The recombinant viruses were serially passaged and their genetic stability was determined with sequencing and titration.

### Evaluation of pathogenicity

Six- to 8-week-old BALB/c mice were randomly divided into two groups (n = 10/group). The mice were challenged with 10^3^ TCID_50_ of either rCVS11-MAB2560 or rCVS11 via the intracranial (i.c.) route. The clinical symptoms, weight change and survival rate of the mice were observed daily within 24 days after infection.

### Immunization in mice and dogs

A pre-experiment was conducted. The immunogen was prepared by mixing inactivated CVS11 with purified MAB2560 protein or PBS. Six- to 8-week-old BALB/c mice were randomly divided into two groups (n = 3/group). The mice were immunized intramuscularly with 100μL of inactivated CVS11 (10^7^ TCID_50_) mixed with PBS, or 100μL of inactivated CVS11 (10^7^ TCID_50_) mixed with MAB2560 protein (50μg). The mice received a total of two immunizations separated by a 2-week interval. Mouse blood was collected at the 1^st^, 2^nd^, 3^rd^, 4^th^ and 5^th^ week following initial immunization.

Then, the inactivated recombinant virus was mixed with Gel02 adjuvant (5:1) to prepare a vaccine. Six- to 8-week-old BALB/c mice were randomly divided into three groups (n = 20/group). The mice were intramuscularly immunized with 10^7^ TCID_50_ of inactivated rCVS11-MAB2560 or rCVS11, with a booster immunization at 2 weeks after the first immunization. The 17- to 21- month-old beagle dogs were randomly divided into two groups (n = 3/group), and intramuscularly immunized with 10^8^ TCID_50_ of inactivated rCVS11-MAB2560 or rCVS11. The booster immunizations were performed twice at 2-week intervals. Mouse (n = 5/group) and dog (n = 3/group) blood was collected at the 1^st^, 2^nd^, 3^rd^, 4^th^, 5^th^, and 6^th^ week after initial immunization. The immunized mice were randomly selected (n = 3/group/day) and the mouse inguinal lymph nodes (ILNs) were collected at 3^rd^, 6^th^, and 9^th^ day post-immunization (dpi). The immunized mice were randomly selected (n = 3/group/day) and mouse spleens were collected at 3^rd^ and 7^th^ week after first immunization.

### Virus-neutralizing antibody (VNA) test

The mouse and dog sera were separated from the collected blood samples. The VNA titers of each serum sample were measured using the fluorescent-antibody virus neutralization (FAVN) test [34]. Briefly, 2-fold dilutions of the heat-inactivated sera were incubated with 100 TCID_50_ of RABV for 1 h at 37°C in 96-well plates. Then, 50μL BHK-21 cells (2×10^5^/mL) were added to each well and the samples were cultured in a 5% CO_2_ incubator at 37°C for 48 h. The cells were fixed with 80% ice-cold acetone, and were then stained with FITC labelled mouse anti-RABV N monoclonal antibody (Melvin, USA). The fluorescent signals were assessed using an inverted fluorescence microscope.

### Activation of bone marrow-derived DCs in vitro

Six- to 8-week old healthy BALB/c mice were euthanized and the femurs were removed. Bone marrow cells were then extracted from the femurs and prepared as previously described [35, 36]. Briefly, the mouse bone marrow was treated with red blood cell lysis buffer for 5 min at RT. The supernatants were discarded after centrifugation at 1200 rpm for 5 min, and the cell pellets were resuspended with RPMI 1640 containing 10% FBS. Bone marrow cells were then cultured in RPMI 1640 supplemented with 10% FBS and 20 ng/mL GM-CSF at a density of 2×10^5^/mL in 24-well plate. After culture for 7 days, the bone marrow-derived DCs were collected and re-cultured in 12 well plates (10^6^/mL) for 2 days, and were then stimulated with rCVS11-MAB2560, or rCVS11, lipopolysaccharide (LPS), or RPMI 1640 for 2 days. Next, the cells were stained with anti-CD11c (PE-Cy7), anti-CD80 (APC), anti-MHC-I (FITC) and anti-MHC-II (PerCP-CY5.5) antibodies. The samples were analyzed using a FACSCalibur flow cytometer (BD Biosciences, USA). For the flow cytometry analysis, the dead cells were excluded from the cell size/granularity parameters according to the FSC/SSC blot.

### Flow cytometry

The collected mouse ILNs were processed to prepare single-cell suspensions as described previously [34]. For the detection of DCs, B cells, T cells, single-cell suspensions were seeded into a 96-well plate with 5x10^5^ cells/well. The cells were then stained with CD11c (PE-Cy7), CD80 (APC), MHC-II (PerCP-CY5.5), MHC-I (FITC), CD4 (FITC), CD8 (PE), CD40 (APC), CD19 (PerCP-Cy5.5), and CD69 (PE-Cy7) antibodies at 4°C for 30 min and examined using a FACSCalibur flow cytometer (BD Biosciences, USA).

The collected mouse spleens were processed to prepare splenocyte cells as described previously [34]. For the detection of immune memory, the splenocyte cells were seeded into a 96-well plate with 2.5×10^5^ cells/well. The cells were then stained with CD4 (FITC), CD8 (PE), CD44 (APC), CD62L (Percy-cy5.5), CD69 (PE-Cy7), and CD19 (PerCP-Cy5.5) antibodies at 4°C for 30 min, and examined using a FACSCalibur flow cytometer (BD Biosciences, USA). For the flow cytometry analysis, the dead cells were excluded from the cell size/granularity parameters according to the FSC/SSC blot.

### ELISPOT

A total of 5×10^5^ splenocyte cells prepared as described above were seeded into the ELISpot plates and were stimulated with the purified rCVS11(10μg/well). After incubation at 37°C for 24 h, the plates were washed five times with PBST, and then incubated with biotin-labeled interferon (IFN)-γ and interleukin (IL)-4 antibodies (1:1000) (provided by the ELISpot kits) for 2h at RT, respectively. The plates were subsequently washed again followed by incubation with streptavidin-conjugated HRP (1:1000) for 1 h at RT. The TMB substrate was then added, and the spot-forming cells (SFCs) in the plates were counted using an ELISpot reader.

### Statistical analysis

Statistical analyses of the data were conducted using a one-way ANOVA in the GraphPad prism software. All the experiments were performed independently at least three times. Error bars represent standard deviations (SD) in each group, as indicated in the figure legends (ns, not significant; *, P <0.05; **, P<0.01; ***, P<0.001).

## Results

### Construction and characterization of rCVS11-MAB2560

**Fig 1:**
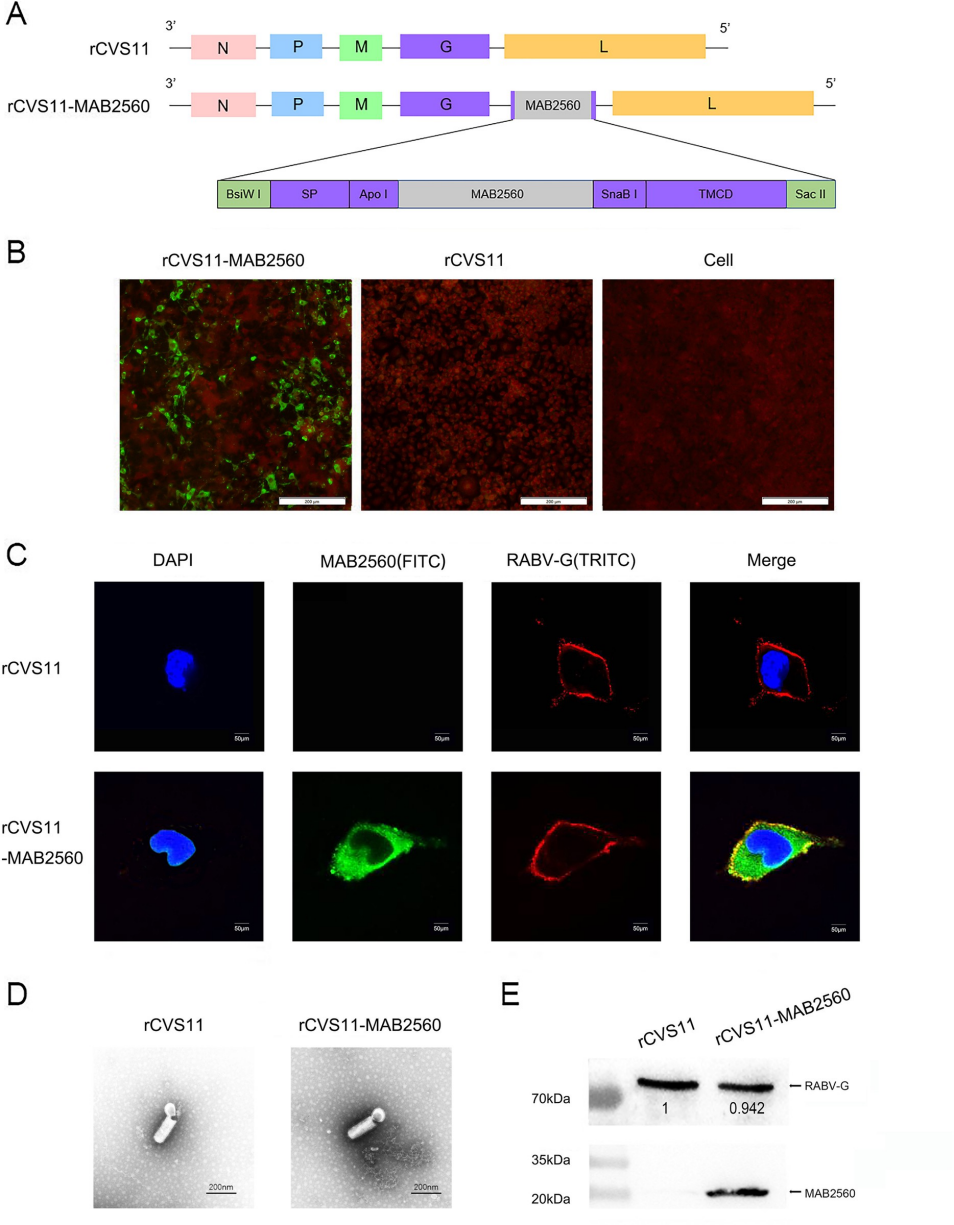
Construction and characterization of rCVS11-MAB2560. (A) Construction diagram of the recombinant plasmid pCDNA3.0-CVS11-SP-MAB2560-TMCD. The *MAB2560* gene was flanked by the signal peptide and the TMCD region from RABV CVS11 G protein, and was inserted into the CVS11 genome between *G* and *L* using the *Bis*W I and *Sac* II restriction sites. (B) NA cells were infected with either the recombinant virus rCVS11-MAB2560 or rCVS11 at 0.1 MOI. After 48h, the cells were incubated with rabbit polyclonal MAB2560 antibody (1:500) as the primary antibody, with FITC-labeled goat anti-rabbit antibody as the secondary antibody (1:500). (C) NA cells were infected with rCVS11-MAB2560 or rCVS11 at 0.4 MOI. After 48h, the cells were incubated with antibodies against MAB2560 (1:200) and G protein (1:500). The nuclei were stained with DAPI. (D) Purified recombinant viruses rCVS11-MAB2560 and rCVS11 were analyzed using electron microscopy (EM) at a magnification of 40000x. (E) The purified viruses were quantified and equal amounts were analyzed with WB, with mouse anti-RABV G mAb (1:500) and rabbit polyclonal MAB2560 antibody (1:200) as the primary antibodies, and HRP-labeled goat anti-mouse IgG (1:10000) and HRP-labeled goat anti-rabbit IgG (1:20000) as the secondary antibodies.

The *MAB2560* gene, linked with the signal peptide and TMCD domain of the RABV CVS11 strain G protein at its 3’ and 5’ ends, respectively, was inserted into the full-length genome between the *G* and *L* genes of the RABV CVS11 strain, to construct the recombinant plasmid pCDNA3.0-CVS11-SP-MAB2560-TMCD (Fig 1A). The recombinant viruses rCVS11-MAB2560 and rCVS11 were rescued in BSR cells by the co-transfection of the recombinant plasmids pCDNA3.0-CVS11-SP-MAB2560-TMCD or pCDNA3.0-CVS11 with helper plasmids. Subsequently, NA cells were infected with either the recombinant virus rCVS11-MAB2560 or rCVS11, and were subjected to IFA and confocal microscopy analysis. The IFA results demonstrated that MAB2560 protein could be successfully expressed by the recombinant virus rCVS11-MAB2560 (Fig 1B). The confocal microscopy results further confirmed that, when cells were not permeabilized, MAB2560 protein could be detected in cytoplasm and membrane of NA cells infected with rCVS11-MAB2560, but not those infected with rCVS11 (Fig 1C). Furthermore, MAB2560 was able to co-localize with RABV G protein in rCVS11-MAB2560-infected cells. The recombinant viruses were then purified and observed with electron microscopy. rCVS11-MAB2560 was found to have similar morphology and size to the parental virus rCVS11 (Fig 1D), indicating that the insertion of the exogenous MAB2560 protein did not affect the spatial structure of recombinant RABV. The WB results demonstrated that the levels of RAVB-G protein expression in rCVS11-MAB2560 were similar to those in rCVS11. Moreover, MAB2560 could be detected with rabbit anti-MAB2560 polyclonal antibody with the expected 23 kDa band in purified recombinant rCVS11-MAB2560 virus, but not in rCVS11 virus (Fig 1E). These results all indicate that the MAB2560 protein is successfully chimerically expressed on the surface of viral particles by the recombinant rCVS11-MAB2560 virus.

### Growth characteristics of rCVS11-MAB2560

**Fig 2:**
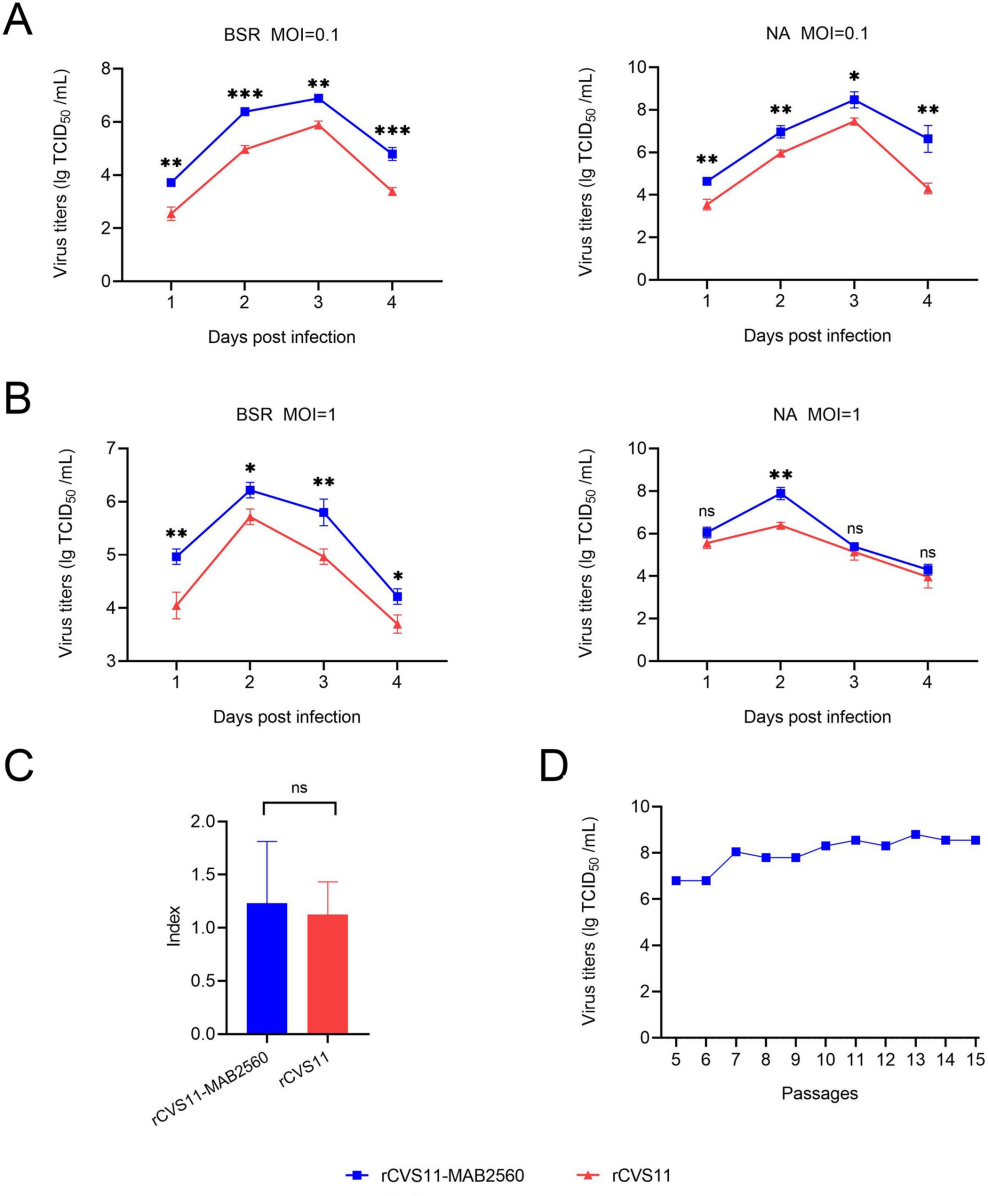
Growth Characteristics of rCVS11-MAB2560. (A and B) Multi-step and one-step growth curves of the recombinant viruses. The recombinant rCVS11-MAB2560 and rCVS11 viruses were separately inoculated into either NA or BSR cells at either MOI = 1 or MOI = 0.1. The cell cultures were collected on the 1^st^, 2^nd^, 3^rd^, and 4^th^ days post-infection, and the viral titer was determined using the FAVN method. (C) Neurotropism of the recombinant virus rCVS11-MAB2560. The viral titers of the recombinant viruses rCVS11-MAB2560 or rCVS11 in NA cells compared to that in BSR cells. All of these experiments in A-C were repeated three times independently and the data were presented as the means ± SD for each group. ns, not significant; *, P<0.05; **, P<0.01. (D) Continual passage of the recombinant viruses, and determination of the viral titers.

To investigate whether the insertion of *MAB2560* gene into the RABV genome would affect the replication of the recombinant rCVS11-MAB2560 virus, the growth curves of both were determined in NA and BSR cells. The results showed that rCVS11-MAB2560 had similar multi-step (Fig 2A) and one-cycle (Fig 2B) growth curves to rCVS11, with higher viral titers, indicating that the chimeric expression of MAB2560 was able to facilitate the viral replication significantly (about 10-fold) in both NA and BSR cells. The results of neural tropism analysis showed that rCVS11-MAB2560 had similar neural tropism to rCVS11 (Fig 2C). The recombinant rCVS11-MAB2560 virus was then passaged continuously, and the viral titers at P5-P15 were stable with the highest titer being 10^8.8^TCID_50_/mL (Fig 2D). The pathogenicity of the recombinant viruses was then evaluated in mice. Compared with the group infected with rCVS11, the clinical symptom score (S1A Fig) and the changes in body weight (S1B Fig) were slightly reduced in the rCVS11- MAB2560 group, while the survival rate improved (S1C Fig), indicating that the insertion of MAB2560 reduced the pathogenicity of rCVS11- MAB2560 in mice.

### rCVS11-MAB2560 induced higher VNA in mice

**Fig 3:**
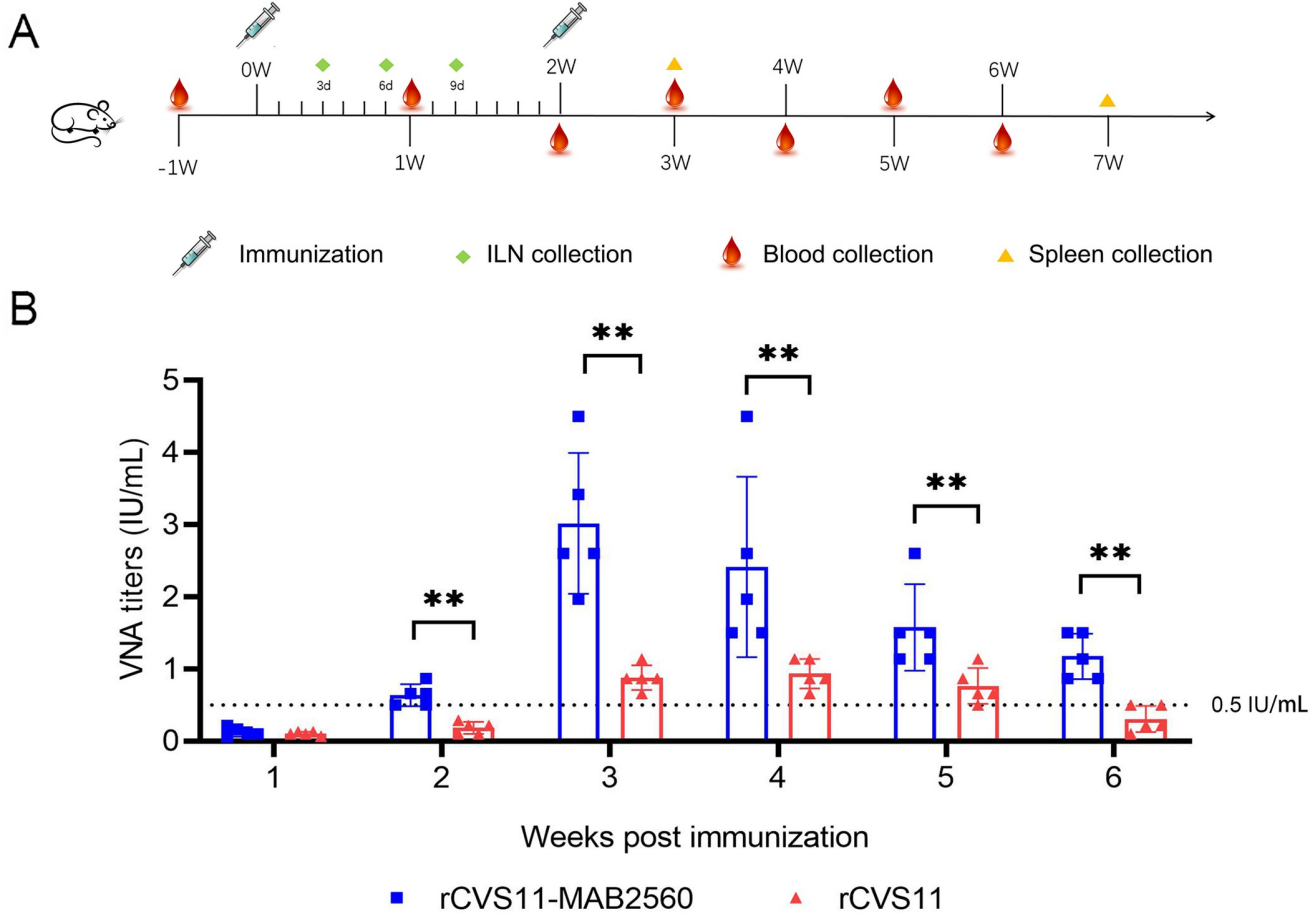
rCVS11-MAB2560 induced stronger VNA in mice than rCVS11. (A) Immunization scheme in mice. Six- to 8- week-old BALB/c mice were randomly divided into three groups (n = 20/group). The mice were immunized intramuscularly with 100μL of inactivated recombinant viruses, or with 100μL of PBS mixed with Gel02 adjuvant. The mice received a total of two immunizations separated by a 2-week interval. ILNs were collected on the 3^rd^, 6^th^, and 9^th^ days post-immunization (dpi), mouse spleens were collected in the 3^rd^ and 7^th^ weeks following the initial immunization, and mouse blood was collected in the 1^st^, 2^nd^, 3^rd^, 4^th^, 5^th^, and 6^th^ weeks following initial immunization. All the clipart used in this figure are quoted from OPENCLIPART (https://openclipart.org/). (B) RABV specific VNAs in mice sera at weeks 1, 2, 3, 4, 5, and 6 post-immunization were measured using a FAVN test. The black dotted line represents the standard 0.5 international unit (IU)/mL level which is recommend as protective neutralizing antibody level by WHO. The data were presented as the means ± SD for each group. **, P<0.01.

We preliminary found that when the MAB2560 protein was mixed with inactivated CVS11 directly, it could increase the production of neutralizing antibodies against RABV in immunized mice (S2 Fig) without clinical adverse effects, indicating that MAB2560 protein could be used as molecular adjuvant with safety. Then, BALB/c mice were immunized intramuscularly with the inactivated recombinant viruses rCVS11-MAB2560 or rCVS11, or with PBS mixed with Gel02 adjuvant, at the indicated time points. Mouse blood, ILNs, and spleen samples were collected at the indicated time points post vaccination for further analysis (Fig 3A). The VNA titer of mouse sera immunized with rCVS11-MAB2560 exceeded 0.5 IU/mL, and was significantly higher than that of the rCVS11 group at the 2^nd^ week following initial vaccination. After booster immunization, the VNA titer of mouse sera in the rCVS11-MAB2560 group significantly increased and was higher than that of rCVS11 group (Fig 3B), indicating that recombinant virus rCVS11-MAB2560 can induce production of neutralizing antibody against RABV in mice faster and at a higher level than rCVS11.

### Activation of DCs by rCVS11-MAB2560 in vitro

**Fig 4:**
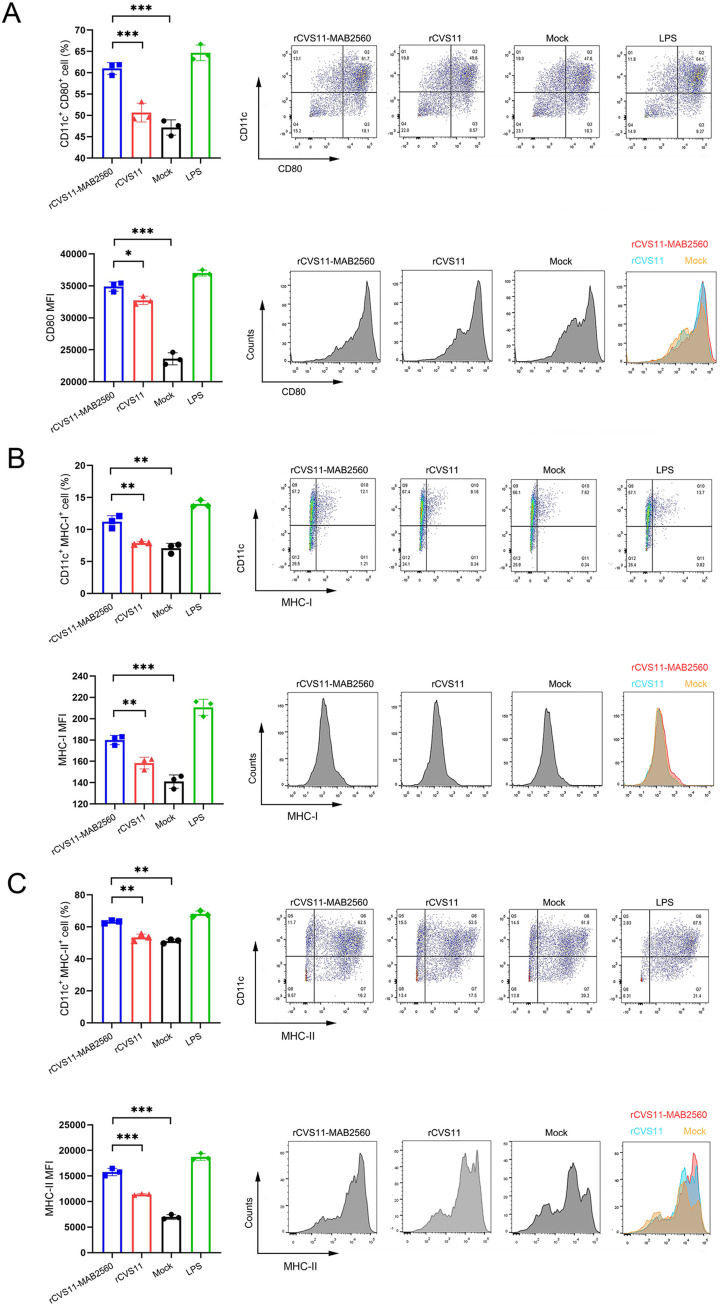
Activation of DCs by rCVS11-MAB2560 in vitro. Bone marrow cells were harvested from healthy BALB/c mice, and DCs were separated and stimulated with the inactivated recombinant viruses rCVS11-MAB2560 and rCVS11. LPS was used as a positive control, and RPMI 1640 (mock) was used as a negative control. Single cell suspensions (10^6^ cells/mL) were stained with antibodies against DC activation markers, and were then analyzed with flow cytometry. (A) The representative flow cytometric plots and percentages of CD11c^+^ CD80^+^ activated DCs (Top) and CD80^+^ MFI (Bottom) in BMDC. (B) The representative flow cytometric plots and percentages of CD11c^+^ MHC-I^+^ activated DCs (Top) and MHC-I^+^ MFI (Bottom) in BMDC. (C) The representative flow cytometric plots and percentages of CD11c^+^ MHC-II^+^ activated DCs (Top) and MHC-II^+^ MFI (Bottom) in BMDC. The data were presented as the means ± SD for each group. ns, not significant; *, P <0.05; **, P<0.01; ***, P<0.001.

The mouse bone marrow-derived DCs were prepared and incubated with inactivated rCVS11-MAB2560 or rCVS11. LPS was used as a positive control and RPMI 1640 (mock) was used as a negative control. Then the DC cells were analyzed by flow cytometric. As expected, the number of CD11c^+^ CD80^+^ (Fig 4A Top), CD11c^+^ MHC-I^+^ (Fig 4B Top), CD11c^+^ MHC-II^+^ (Fig 4C Top) double-positive cells was significantly higher in the rCVS11-MAB2560 group than in the rCVS11 and mock groups. At the same time, we conducted the mean fluorescence intensity (MFI) analysis of CD80 (Fig 4A Bottom), MHC-I (Fig 4B Bottom) and MHC-II (Fig 4C Bottom) on CD11c positive cells, and found that the MFI of double positive cells of rCVS11-MAB2560 group was significantly higher than that of rCVS11 group and Mock. The results showed that rCVS11-MAB2560 could activate DCs in vitro.

### Recruitment of DCs by rCVS11-MAB2560 in vivo

**Fig 5:**
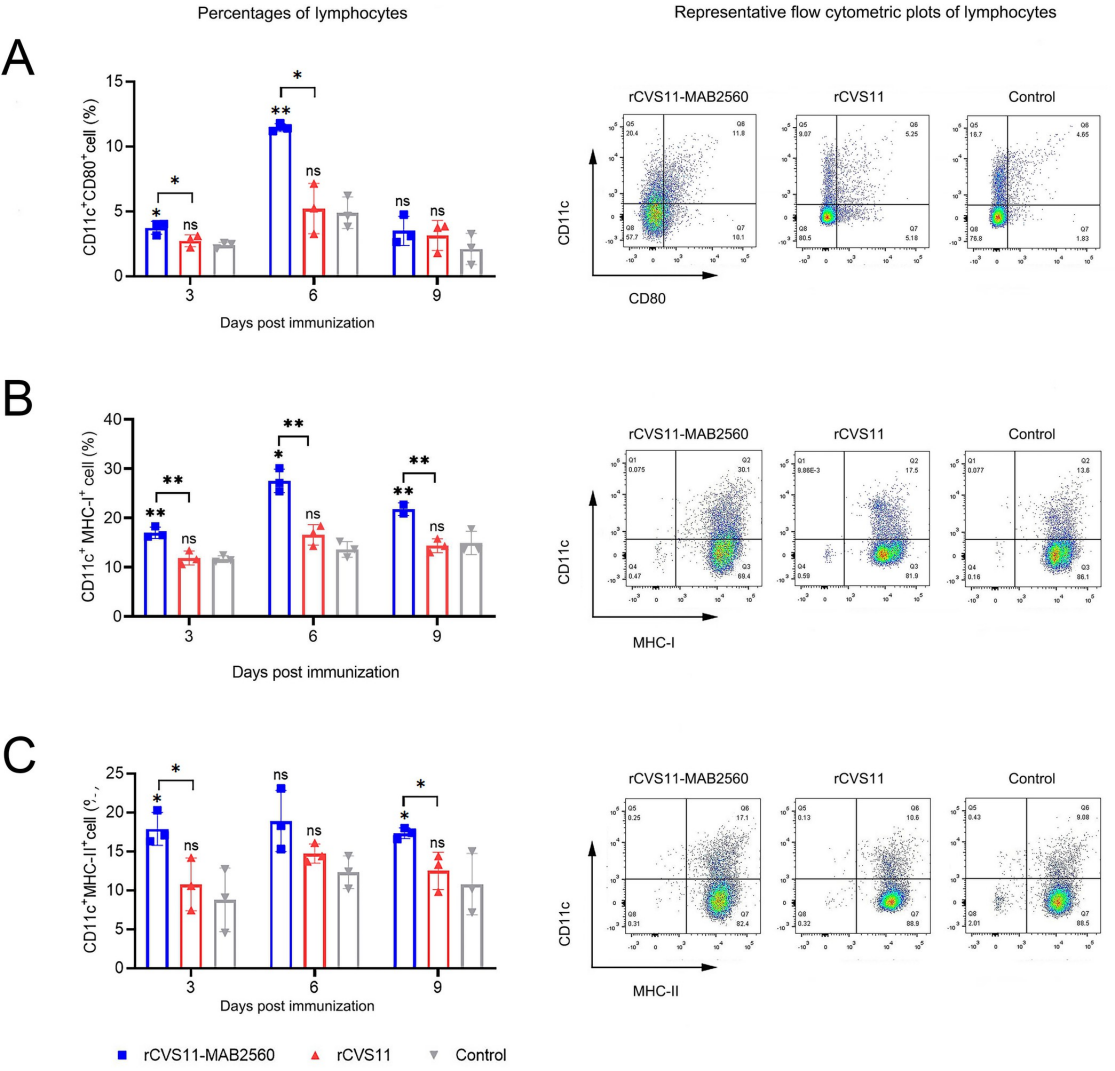
Recruitment of DCs by rCVS11-MAB2560 in vivo. On the 3^rd^, 6^th^ and 9^th^ dpi, ILNs were collected from the vaccinated BALB/c mice and single cell suspensions (10^6^ cells/mL) were prepared. The single cell suspensions were stained with antibodies against DC activation markers and analyzed with flow cytometry. (A) The representative flow cytometric plots (6^th^ dpi) and percentages of CD11c^+^ and CD80^+^ activated DCs in the ILNs of immunized mice. (B) The representative flow cytometric plots (6^th^ dpi) and percentages of CD11c^+^ and MHC-I^+^ activated DCs in the ILNs of immunized mice. (C) The representative flow cytometric plots (3^rd^ dpi) and percentages of CD11c^+^ and MHC-II^+^ activated DCs in the ILNs of immunized mice. The data were presented as the means ± SD for each group. ns, not significant; *, P <0.05; **, P<0.01; ***, P<0.001.

The ability of inactivated rCVS11-MAB2560 to recruit and/or activate DCs was then evaluated in mice. ILNs from immunized mice were collected at 3^rd^, 6^th^ and 9^th^ dpi, and single cell suspensions were prepared for the detection of activated DCs in ILNs (CD11c^+^ and CD80^+^, MHC-I^+^, MHC-II^+^) with flow cytometry. Compared to the rCVS11 group and control group, the rCVS11-MAB2560 group recruited more DC cells, the number of CD11c^+^ CD80^+^ (Fig 5A) double positive cells increased significantly at 3^rd^ and 6^th^ dpi, and the number of CD11c^+^ MHC-I^+^ (Fig 5B) double positive cells increased significantly at 3^rd^, 6^th^ and 9^th^ dpi, CD11c^+^ MHC-II^+^ (Fig 5C) double positive cells increased significantly at 3^rd^ and 9^th^ dpi. The inactivated rCVS11 could not recruit DCs in vivo, with no increase in the number of CD11c^+^ positive cells and CD11c^+^ CD80^+^ (Fig 5A), CD11c^+^ MHC-I^+^ (Fig 5B), or CD11c^+^ MHC-II^+^ (Fig 5C) double-positive cells, indicating that inactivated rCVS11-MAB2560 could more effectively increase the number of activated DCs in ILNs than inactivated rCVS11 at the early stage of immunization in vivo.

### Recruitment of T and B cells by inactivated rCVS11-MAB2560

**Fig 6:**
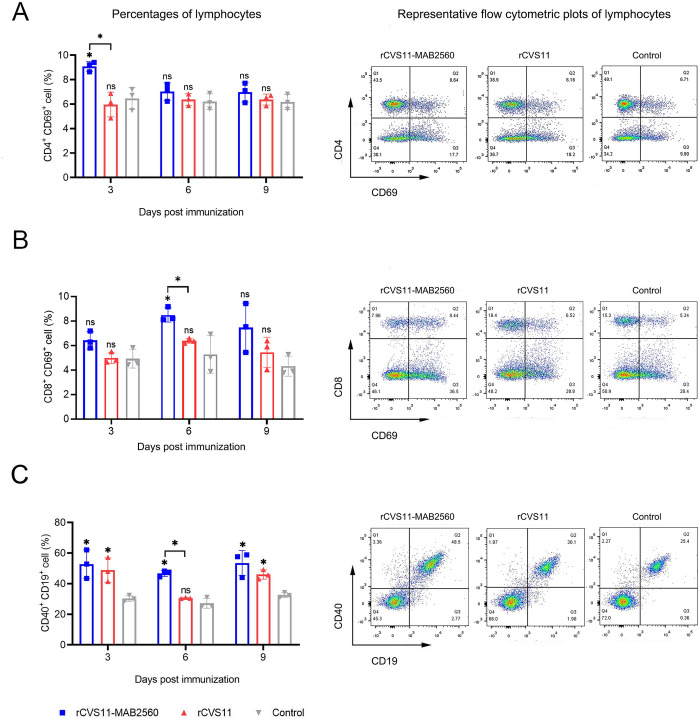
Recruitment of T and B cells by rCVS11-MAB2560. On the 3^rd^, 6^th^ and 9^th^ dpi, ILNs were collected from the vaccinated BALB/c mice and single cell suspensions (10^6^ cells/mL) were prepared. The suspensions were stained with antibodies against T and B cell activation markers and were analyzed using flow cytometry. (A) The representative flow cytometric plots (3^rd^ dpi) and percentages of CD4^+^ and CD69^+^ recruited and/or activated CD4^+^ T cells in the ILNs of immunized mice. (B) The representative flow cytometric plots (6^th^ dpi) and percentages of CD8^+^ and CD69^+^ recruited and/or activated CD8^+^ T cells in the ILNs of immunized mice. (C) The representative flow cytometric plots (6^th^ dpi) and percentages of CD40^+^ and CD19^+^ recruited and/or activated B cells in the ILNs of immunized mice. The data were presented as the means ± SD for each group. ns, not significant; *, P <0.05.

The activation and proliferation of DCs enhances their ability to present antigens, thereby promoting the recruitment and/or activation of T and then B cells. To investigate whether the recombinant virus rCVS11-MAB2560 recruits and/or activates T and B cells in the early stages of immunization, the recruitment and/or activation of T and B cells in ILNs harvested on the 3^rd^, 6^th^ and 9^th^ dpi was analyzed. The results showed that compared with rCVS11, inactivated rCVS11-MAB2560 recruited more T cells at 3^rd^ and 6^th^ dpi, at the same time, the number of CD4^+^ CD69^+^ double positive cells increased at 3^rd^ dpi (Fig 6A), and CD8^+^ CD69^+^ double positive cells increased at 6^th^ dpi (Fig 6B). Both inactivated rCVS11-MAB2560 and rCVS11 could induce the recruitment of B cells and increase the number of activated B cells at 3^rd^, 6^th^, and 9^th^ dpi (Fig 6C).

Compared with rCVS11 group, the number of activated B cells in rCVS11-MAB2560 group increased significantly at 6^th^ dpi (Fig 6C), after the recruitment of T cells (Fig 6A and 6B), indicating that rCVS11-MAB2560 promoted the recruitment of T and B cells in mice.

### Production of T helper 1 (Th1) and T helper 2 (Th2) cytokines by rCVS11-MAB2560

**Fig 7:**
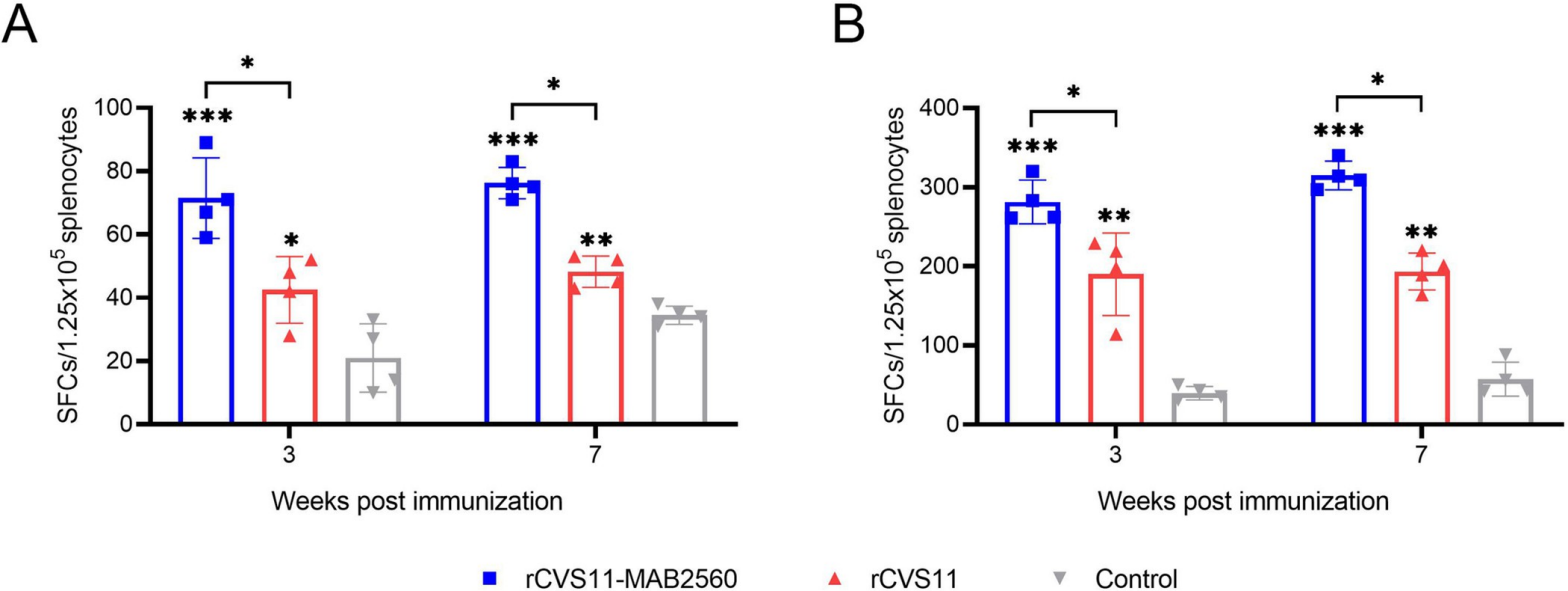
Induction of Th1 and Th2 cytokine production by rCVS11-MAB2560. Mouse spleens were collected at the 3^rd^ and 7^th^ week following initial immunization, and were prepared into splenocyte suspensions. RABV-specific IFN-γ (A) or IL-4 (B) SFCs were quantified using ELISpot assays. The data were presented as the means ± SD for each group. *, P <0.05; **, P<0.01; ***, P<0.001.

To investigate the effect of rCVS11-MAB2560 on the Th1 and Th2 immune responses, the secretion of Th1 and Th2 cytokines by the spleens of mice immunized with recombinant viruses was analyzed at the 3^rd^ and 7^th^ week post-immunization. As expected, compared with the rCVS11 group, more IFN-γ (Fig 7A) and IL-4 (Fig 7B) SFCs were detected in the spleens of mice immunized with rCVS11-MAB2560, indicating that the recombinant virus rCVS11-MAB2560 could simultaneously enhance the production of Th1 and Th2 cytokines after immunization.

### rCVS11-MAB2560 induced good immune memory after immunization

**Fig 8:**
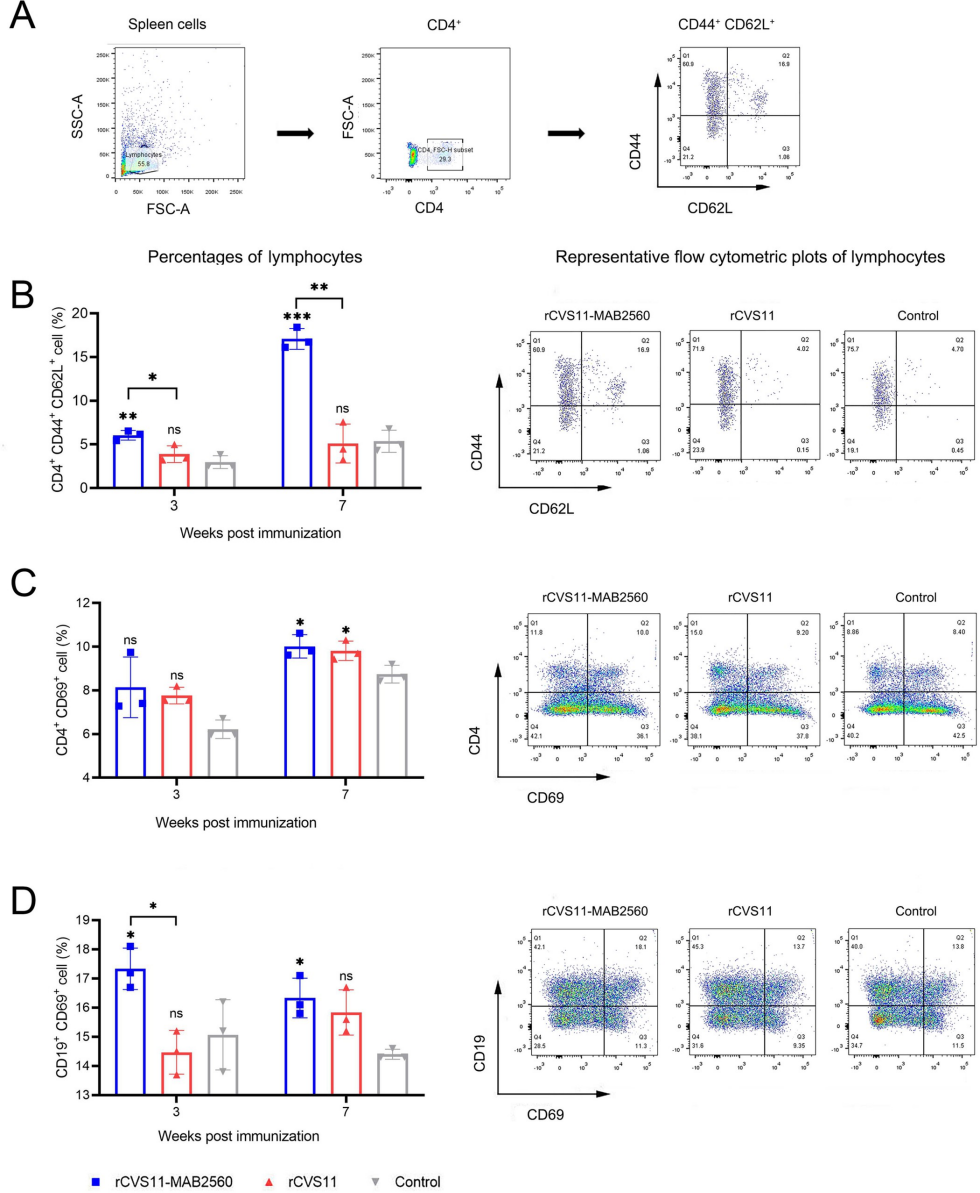
rCVS11-MAB2560 induced good immune memory after immunization in mice. At the 3^rd^ and 7^th^ weeks following initial immunization, spleens were collected from the vaccinated BALB/c mice. Single cell suspensions of splenocyte cells (10^6^ cells/mL) were stained with antibodies against TCM, T and B cell activation markers, and were analyzed with flow cytometry. (A) Flow gate strategy of CD4^+^ TCM (7^th^ week post immunization). (B) The representative flow cytometric plots (7^th^ week post immunization) and percentages of CD4^+^, CD44^+^ and CD62L^+^ recruited and/or activated CD4^+^ T cells in the spleens of immunized mice. (C) The representative flow cytometric plots (7^th^ week post immunization) and percentages of CD4^+^, CD69^+^ recruited and/or activated CD4^+^ T cells in the spleens of immunized mice. (D) The representative flow cytometric plots (3^rd^ week post immunization) and percentages of CD19^+^ and CD69^+^ recruited and/or activated B cells in the spleens of immunized mice. The data were presented as the means ± SD for each group. ns, not significant; *, P <0.05; **, P<0.01.

Immunological memory induced by vaccination is critical in host resistance against pathogen re-infection [37]. When the host is subsequently infected with the same pathogen, central memory T cells (TCM) induced by vaccination are recruited and/or activated and are differentiated into CD4^+^ and CD8^+^ T cells, which in turn secrete cytokines to recruit and/or activate B cells to product neutralizing antibodies. To detect the immunological memory induced by the recombinant viruses in mice, splenic lymphocytes were isolated from the immunized mice at the 3^rd^ and 7^th^ weeks after the first immunization. The splenic lymphocytes were stimulated with the purified rCVS11-MAB2560 and analyzed using flow cytometry. The gating strategy was shown in Fig 8A. The results showed that compared to control group, the inactivated rCVS11-MAB2560 recruited more CD4^+^ TCM (CD4^+^, CD44^+^, and CD62L^+^) at the 3^rd^ week post initial immunization, and the number of CD4^+^ TCM increased continuously to a maximum at the 7^th^ week (Fig 8B). More CD4^+^ T cells (CD4^+^, CD69^+^) were recruited at the 7^th^ week (Fig 8C) and more B cells (CD19^+^, CD69^+^) at 3^rd^ and 7^th^ week following the initial immunization (Fig 8D). Moreover, the inactivated rCVS11-MAB2560 recruited more CD4^+^ TCM (Fig 8B) at 3^rd^ and 7^th^ week and more B cells (Fig 8D) at 3^rd^ week after the first immunization than did rCVS11. However, the numbers of CD8^+^ TCM (S3A Fig) and CD8^+^ T cells (S3B Fig) in the spleens of mice immunized with rCVS11-MAB2560 were not enhanced, and were similar to those in the rCVS11 or control groups. In summary, mice immunized with the recombinant virus rCVS11-MAB2560 developed a higher level of immune memory, which may be able to more rapidly produce the VNAs necessary to resist the re-infection with RABV than those vaccinated with rCVS11.

### rCVS11-MAB2560 induced stronger VNA in dogs

**Fig 9:**
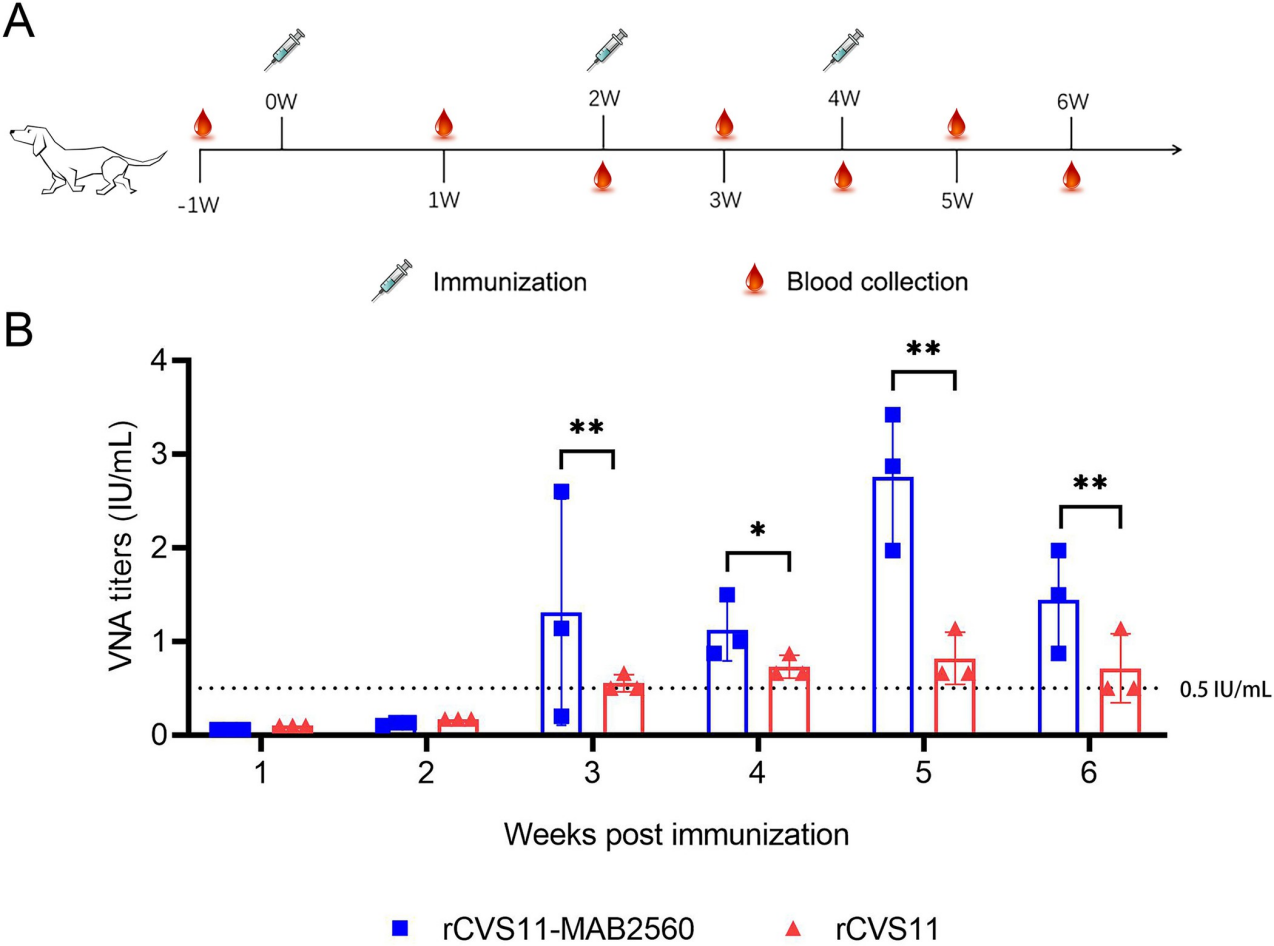
rCVS11-MAB2560 induces stronger VNA in dogs than rCVS11. (A) Immunization scheme in dogs. Beagle dogs (17- to 21- months-old) were randomly divided into two groups (n = 3/group). The dogs were immunized intramuscularly with 1mL of inactivated rRABVs (10^8^ TCID_50_/dog), or with 1mL of PBS mixed with Gel02 adjuvant. The dogs received three immunizations in total, at 2-week intervals. Dog blood was collected the 1^st^, 2^nd^, 3^rd^, 4^th^, 5^th^, and 6^th^ week following the initial immunization. All the clipart used in this figure are quoted from OPENCLIPART (https://openclipart.org/). (B) RABV-specific VNAs in dog sera at weeks 1, 2, 3, 4, 5, and 6 post-immunization were measured using a FAVN test. The black dotted line represents the standard 0.5 international unit (IU)/mL level which is recommend as protective neutralizing antibody level by WHO. The data were presented as the means ± SD for each group. *, P <0.05; **, P<0.01.

We further tested the immunogenicity of rCVS11-MAB2560 in dogs. The immunization programs proceeded as illustrated in the schematic diagram (Fig 9A). The VNA titer in the serum samples from the rCVS11-MAB2560 vaccinated dogs was detectable at the first week post-immunization, and significantly increased following the booster immunization (Fig 9B). Meanwhile, the titer of VNAs induced by the inactivated rCVS11-MAB2560 was significantly higher than that induced by rCVS11.

## Discussion

The key to developing a new generation of highly effective rabies vaccines is promoting the ability to rapidly activate host immune response to produce high levels of neutralizing antibodies. It has been demonstrated that cytokines and chemokines can be used as adjuvants to enhance the immune effect of vaccine by promoting the activation of DCs and B cells [38–40], which suggested to us that incorporating a cytokine into RABV could be a new strategy to improve host immune responses to a vaccine. Development of the new generation of rabies vaccines has mainly focused on inactivated vaccines, however, inactivated vaccines cannot replicate in the host. Chimeric expression of molecular adjuvants on the surface of RABV particles is therefore a good choice, and can mean that the recombinant virus can promote the host immune response even in an inactivated state. Previous studies have demonstrated that an inactive chimeric recombinant RABV expressing a DC-binding peptide is able to induce a robust VNA response after vaccination, and leads to better protection against lethal virus challenge than the recombinant virus without the DC-binding peptide [41]. Another study constructed a recombinant virus in which the B cell activating factor (BAFF), a member of the tumor necrosis factor (TNF) superfamily of cytokines that links innate and adaptive immunities, was incorporated into the membrane of the virus particles. The BAFF boosted the speed and magnitude of vaccine-induced antibody responses [42]. This kind of recombinant virus exhibits huge potential as a safer and more effective inactivated rabies vaccine. Here, we constructed a recombinant virus, rCVS11-MAB2560, which chimerically expressed MAB2560 protein (a DC-targeting molecular) on the surface of the RABV particles (Fig 1). Expression of G protein, the major immunogen of RABV, was not reduced by co-expression with MAB2560 protein in rCVS11-MAB2560, allowing the recombinant virus to maintain the original immunogenicity of RABV. Moreover, compared to rCVS11, the recombinant virus rCVS11-MAB2560 could achieve higher viral titers in both NA and BSR cells (Fig 2), but had a lower pathogenicity in mice (S1 Fig). This may have been because the exogenous expression of MAB2560 by rCVS11-MAB2560 triggered a stronger immune response in mice than rCVS11, that accelerated the elimination of the virus from the body, resulting in a reduction of clinical symptoms. The mechanisms underlying the improved replication of rCVS11-MAB2560 in NA and BSR cells compared with the parent virus needs to be further investigated. However, these characteristics give the recombinant virus an advantage in subsequent vaccine production, with reduced costs and increased safety.

Subsequently, we found that the inactivated recombinant virus rCVS11-MAB2560 induced the production of VNAs to a higher level than the parent virus, and the protective titers were rapidly achieved in both mice and dogs (Figs 3 and 9), which could provide effective protection against RABV infection earlier post vaccination. Our further studies confirmed that compared with the parent virus rCVS11, the recombinant virus rCVS11-MAB2560 efficiently activated more bone marrow-derived DCs in vitro and recruited more DCs in ILNs (the secondary lymphoid organ) from vaccinated mice at the early stages following the initial immunization (Figs 4 and 5). DCs are the main professional antigen-presenting cells (APCs) and the only APCs that can stimulate naive T lymphocytes, therefore playing an important role in activating the host immune response [43]. Our results suggested that the MAB2560 protein chimerically expressed on the viral particles of the recombinant virus rCVS11-MAB2560 helped to promote the host immune response by enhancing the recruitment of DCs, which is consistent with a previous study [32]. In addition, our results showed that the total numbers of MHC-I and MHC-II positive DCs were simultaneously enhanced in mice immunized with rCVS11-MAB2560, but not in those vaccinated with rCVS11 (Figs 4 and 5). Both MHC-I and MHC-II can detect exogenous antigens and present antigens on the surface of DCs, which are then recognized by CD8^+^ and CD4^+^ T cells to induce the comprehensive immune responses [44, 45]. As expected, our results demonstrated that, compared with rCVS11, the inactivated rCVS11-MAB2560 can trigger earlier and stronger T cell and B cell responses in mice (Fig 6), which could promote the production of VNAs in mice, as illustrated in Fig 3.

Moreover, the intensity of the Th1 response to the inactivated rCVS11-MAB2560 virus was significantly increased compared to that in the inactivated rCVS11 group (Fig 7), indicating that MAB2560 protein expressed by rCVS11-MAB2560 played an important role in inducing host immune response as an effective Th1 polarizing adjuvant [32]. A strong Th 1 response can provide better protection against RABV infection because the Th1 immune response is a potent inducer of anti-viral effector functions [46, 47]. Meanwhile, compared to the inactivated rCVS11 group, the group exposed to the recombinant virus rCVS11-MAB2560 showed that more effector T cells had been stimulated to differentiate into CD4^+^ memory T cells, which helps the host acquire the ability to mount a secondary response quickly upon subsequent viral antigen exposure (Fig 8). All these results indicate that the recombinant virus rCVS11-MAB2560 has better immunogenicity than rCVS11. MAB2560 protein is an effective molecular adjuvant when displayed on the surface of recombinant viral particles, and facilitates host cellular and humoral immune responses by targeting and recruiting DCs. The molecular mechanism of MAB2560 protein as an adjuvant to boost host immunity needs to be further illustrated by co-culturing the MAB2560-treated DCs with naive T cells in vitro, or quantifying the CD4 and CD8 T cells, DCs, and B cells in immunized mouse ILNs and spleens using immunofluorescent microscopy, to give insights into the phenotypes of T cells that these naive T cells differentiate into and the migrated spatial information of these immune cells induced by MAB2560.

In summary, the recombinant rabies virus rCVS11-MAB2560 we constructed here can induce a more efficient immune response than the parent virus in both mice and dogs through recruitment of DCs and activating the subsequent signal pathway. This suggests that rCVS11-MAB2560 has potential as a safe and efficient inactivated rabies vaccine candidate. Given that adjuvants play an indispensable role in the development of modern vaccines, better adjuvants with fewer side effects should be sought in the future to improve the immune effectiveness of vaccines.

## Supporting information

S1 Fig Pathogenicity of rCVS11-MAB2560. Six- to 8-week-old BALB/c mice (n = 10/group) were challenged with 10^3^ TCID_50_ recombinant virus, either rCVS11-MAB2560 or rCVS11. The clinical symptom score (A), changes of body weight (B), and the survival rate (C) of mice were measured.
(TIF)

S2 Fig Immunization of mice with a mixture of MAB2560 and CVS11 induced higher levels of VNA. Six- to 8- week-old BALB/c mice were randomly divided into two groups (n = 3/group). The mice were immunized intramuscularly with 100μL of inactivated CVS11 (10^7^ TCID_50_) mixed with PBS, or with 100μL of CVS11 (10^7^ TCID_50_) mixed with MAB2560 protein (50μg). The mice received a total of two immunizations separated by a 2-week interval and mouse blood was collected in the 1^st^, 2^nd^, 3^rd^, 4^th^ and 5^th^ weeks following initial immunization. RABV specific VNAs in mouse sera were measured using a FAVN test. The data were presented as the means ± SD for each group. *, P <0.05; **, P<0.01.
(TIF)

S3 Fig CD8^+^ TCM cells and CD8^+^ T cells were not induced by the recombinant viruses. At the 3^rd^ and 7^th^ week after the initial immunization, spleens were collected from the vaccinated BALB/c mice and single cell suspensions (10^6^ cells/mL) were stained with antibodies against TCM and T cell activation markers. Samples were analyzed with flow cytometry. (A) The percentages of CD8^+^, CD44^+^, and CD62L^+^ recruited CD8^+^ TCM cells in the spleens of immunized mice. (B) The percentages of CD8^+^ and CD69^+^ recruited CD8^+^ T cells in the spleens of immunized mice. The data were presented as the means ± SD for each group. ns, not significant.
(TIF)
