# Supplementary figures and images for: A recombinant rabies virus chimera expressing the DC-targeting molecular MAB2560 shows enhanced vaccine immunogenicity through activation of dendritic cells
Source: PLoS Negl Trop Dis. 2023 Apr 24;17(4):e0011254. doi: 10.1371/journal.pntd.0011254 (PMC10124880; doi:10.1371/journal.pntd.0011254)

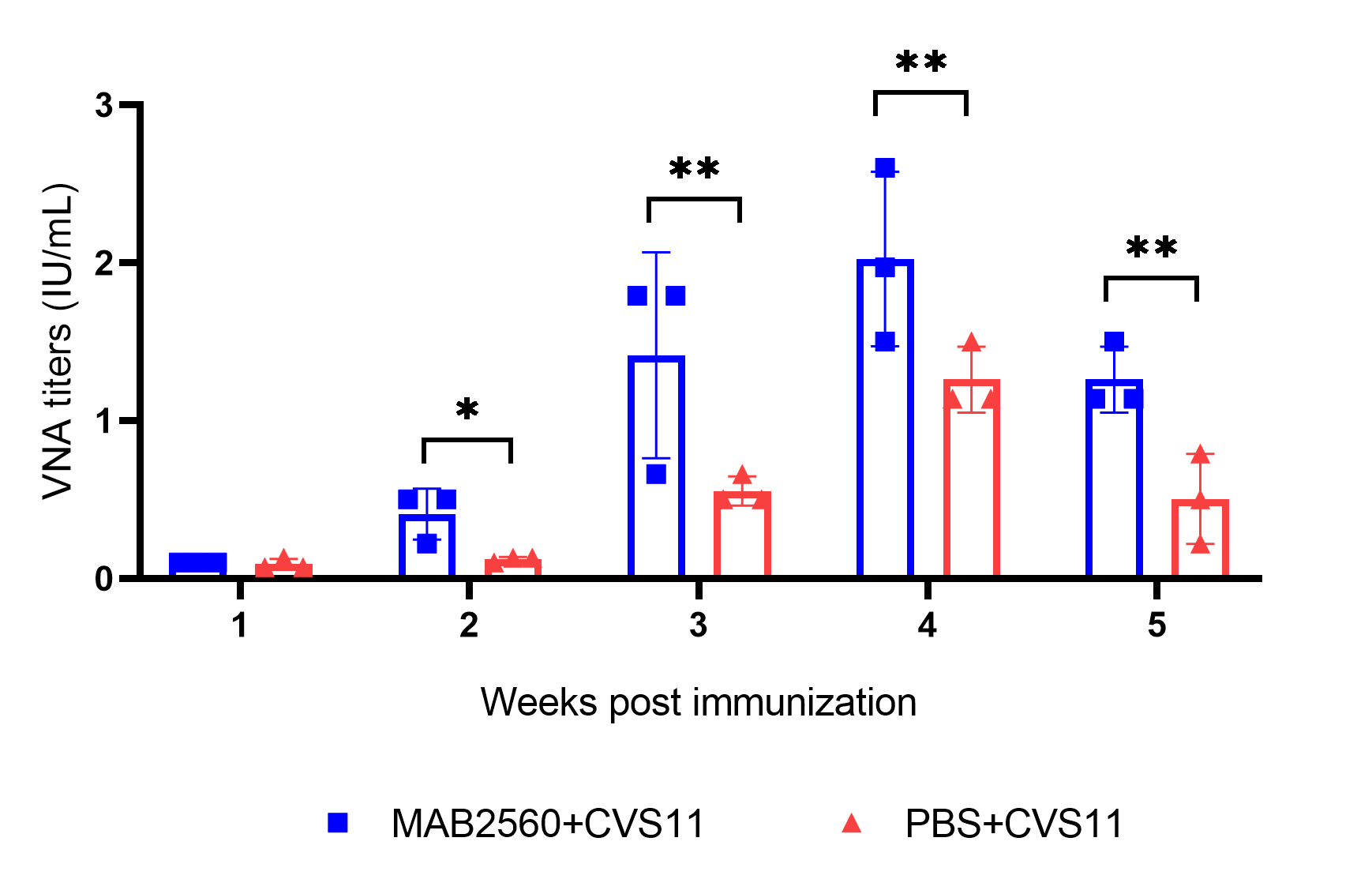

Supplement: S2 Fig — Six- to 8- week-old BALB/c mice were randomly divided into two groups (n = 3/group). The mice were immunized intramuscularly with 100μL of inactivated CVS11 (107 TCID50) mixed with PBS, or with 100μL of CVS11 (107 TCID50) mixed with MAB2560 protein (50μg). The mice received a total of two immunizations separated by a 2-week interval and mouse blood was collected in the 1st, 2nd, 3rd, 4th and 5th weeks following initial immunization. RABV specific VNAs in mouse sera were measured using a FAVN test. The data were presented as the means ± SD for each group. *, P <0.05; **, P<0.01. (TIF) [file pntd.0011254.s002.tif]
